# Dorsal Column Nuclei Neural Signal Features Permit Robust Machine-Learning of Natural Tactile- and Proprioception-Dominated Stimuli

**DOI:** 10.3389/fnsys.2020.00046

**Published:** 2020-07-28

**Authors:** Alastair J. Loutit, Jason R. Potas

**Affiliations:** ^1^School of Medical Sciences, University of New South Wales Sydney, Kensington, NSW, Australia; ^2^The Eccles Institute of Neuroscience, John Curtin School of Medical Research, Australian National University, Canberra, ACT, Australia

**Keywords:** feature learnability, neural prosthesis, supervised back-propagation artificial neural network, brain-machine interface, cuneate, gracile

## Abstract

Neural prostheses enable users to effect movement through a variety of actuators by translating brain signals into movement control signals. However, to achieve more natural limb movements from these devices, the restoration of somatosensory feedback is required. We used feature-learnability, a machine-learning approach, to assess signal features for their capacity to enhance decoding performance of neural signals evoked by natural tactile and proprioceptive somatosensory stimuli, recorded from the surface of the dorsal column nuclei (DCN) in urethane-anesthetized rats. The highest performing individual feature, spike amplitude, classified somatosensory DCN signals with 70% accuracy. The highest accuracy achieved was 87% using 13 features that were extracted from both high and low-frequency (LF) bands of DCN signals. In general, high-frequency (HF) features contained the most information about peripheral somatosensory events, but when features were acquired from short time-windows, classification accuracy was significantly improved by adding LF features to the feature set. We found that proprioception-dominated stimuli generalize across animals better than tactile-dominated stimuli, and we demonstrate how information that signal features contribute to neural decoding changes over the time-course of dynamic somatosensory events. These findings may inform the biomimetic design of artificial stimuli that can activate the DCN to substitute somatosensory feedback. Although, we investigated somatosensory structures, the feature set we investigated may also prove useful for decoding other (e.g., motor) neural signals.

## Introduction

Neural prostheses enable users to control robotic limbs, computer cursors, or even effect movement of the users own limbs, by translating brain signals into movement control signals (Ethier et al., 2012; Hochberg et al., 2012; Collinger et al., 2013; Gilja et al., 2015; Jarosiewicz et al., 2015; Bouton et al., 2016; Capogrosso et al., 2016; Ajiboye et al., 2017). Currently, neural prosthetic performance is still poor compared to natural limb movements, particularly for dexterous object manipulation. However, motor control performance can be significantly improved by restoring somatosensory feedback that rapidly updates limb status.

Several groups have stimulated monkey or human somatosensory cortex to evoke percepts such as vibration, pressure, and stimulus location, and have shown that this somatosensory feedback improves dexterous manipulation capabilities of motor neural prostheses (O’Doherty et al., 2009, 2019; Tabot et al., 2013; Klaes et al., 2014; Kim et al., 2015; Flesher et al., 2016, 2019; Salas et al., 2018). However, we have recently suggested that the dorsal column nuclei (DCN) and their associated external cuneate nuclei, nuclei X, and Z (DCN-complex) have anatomical advantages over the cortex as a sensory neural prosthesis target (Loutit and Potas, 2020a; Loutit et al., 2020). Activating the DCN-complex with a neural prosthesis capable of mimicking natural somatosensory signals has the potential to simultaneously inform not only the cortex but several other regions essential for motor control, including the cerebellum and tegmentum that are bypassed by a cortical neural prosthesis.

While the DCN has begun to receive attention as a neural prosthetic target, microelectrode arrays have been chronically implanted in macaque DCN (Richardson et al., 2015; Sritharan et al., 2016; Loutit et al., 2017, 2019; Suresh et al., 2017; Loutit and Potas, 2020a). To date, these DCN rigid chronic electrode array implants have resulted in some failures due to head and neck movements damaging the wire bundles or dislodging the array from the brain tissue (Suresh et al., 2017). However, rapidly advancing soft implantable electrode technologies, such as microelectrode “threads” that can be sewn into brain tissue at densities of approximately 3,000 electrodes/25 mm^2^ (Musk and Neuralink, 2019), present a promising solution for targeting the DCN (Loutit and Potas, 2020a). Yet, knowledge of DCN neurophysiology and how to effectively activate DCN neural populations to restore somatosensory feedback is limited. Some state-of-the-art peripheral somatosensory neural prostheses use biomimetic stimulus patterns to mimic attributes of fast- or slowly-adapting afferents, by modulating stimulus frequency or amplitude at the onset, offset, static or dynamic phases of stimuli (Valle et al., 2018; George et al., 2019). These biomimetic stimulus patterns were reported to elicit more naturalistic sensations and improved object discrimination performance, compared to linear feedback algorithms that simply increase stimulus intensity (amplitude or frequency) with increased force applied to a robotic force sensor. To construct such biomimetic stimulus patterns for the DCN, we first need to determine which properties of DCN neural activity are most relevant to somatosensory stimuli from which they were evoked.

We previously devised a metric, which we termed *feature-learnability*, for quantifying information relevance of DCN neural signal features for discriminating somatosensory stimuli (Loutit et al., 2019). Using feature-learnability, we demonstrated that electrical stimulation of peripheral afferents results in robust and reproducible DCN neuronal activity, within and across animals. This suggests that different animals undergo the same DCN neural processes in response to an electrically-evoked peripheral event. But how reproducible is DCN activity under conditions of naturally presented stimuli? In the present study, we used feature-learnability to assess a battery of DCN neural signal features for their information content about the natural peripheral stimuli from which they were evoked. Natural stimuli consist of a degree of mixed tactile and proprioceptive events; i.e., some tactile stimuli generate digit movement, and it is not possible to move a joint without distorting the skin around that joint. To study how tactile and proprioceptive information is captured by DCN signal features and how consistent this is across animals, we contrived tactile- and proprioceptive-dominant stimuli, whilst recording somatosensory-evoked DCN signals using a surface multielectrode array (sMEA). These stimuli are not exclusively tactile or proprioceptive, but rather, they mainly recruit one type of afferent whilst minimizing recruitment of the other.

In the present study, we aimed to explore the feature-learnability of DCN somatosensory potentials evoked by tactile- and proprioceptive-dominant stimuli. We use feature-learnability to evaluate a diverse set of neural signal features for information content relevant to decoding mechanically evoked tactile- and proprioception-dominated stimuli. From somatosensory-evoked DCN neural signals, we extracted 22 features from four categories: two categories, high-frequency (HF) and low-frequency (LF), were derived from time-domain signals, and the remaining two categories were derived from frequency-domain features; HF power spectral density (HF PSD), and LF power spectral density (LF PSD) features. As signal features represent underlying neuronal processes, high feature-learnability indicates the presence of a robust and reproducible neuronal process for natural stimuli. Furthermore, high feature-learnability across animals indicates that a similar neural process is present across different animals. How feature-learnability changes as a function of time throughout a dynamic stimulus indicates the changing importance of that feature to a peripheral event during different phases of its progression, and therefore is valuable for future biomimetic stimulation applications. While our primary focus was to investigate features of somatosensory DCN signals, the feature set and our approach to determining the most information-rich features for decoding neural signals may provide insight into other neural signal decoding applications, such as motor systems.

## Materials and Methods

### Animals

We used 8-week-old male Wistar rats (283–464 g; *n* = 6; Australian Phenomics Facility, Canberra, ACT, Australia). There were 1–3 animals housed per cage, with a 12/12-h light/dark cycle. Food and water were available for animals to access *ad libitum*. All procedures were approved by the Australian National University Animal Experimentation Ethics Committee (A2014/52) and adhered to the Australian code of practice for the care and use of animals for scientific purposes.

### Surgery

**Figure 1:**
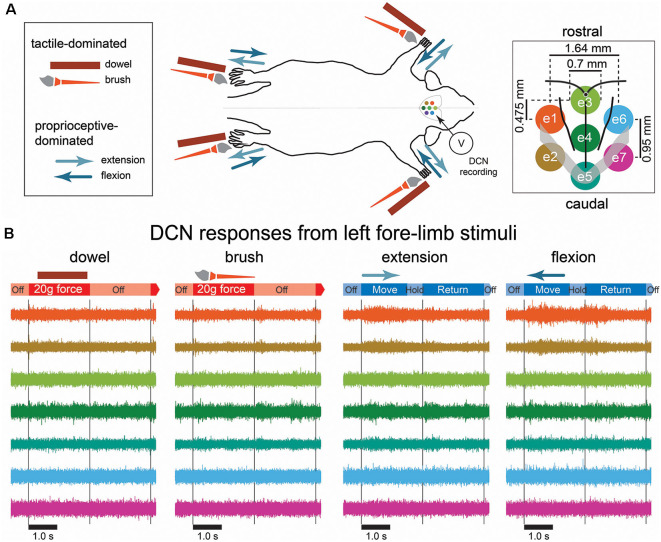
Mechanical stimulation conditions, recording arrangement, and example signals recorded from the dorsal column nuclei (DCN). **(A)** A schematic diagram of the mechanical stimulation and surface array recording paradigm. Each of the four limbs was individually stimulated with a mechanical stimulus as shown in the left insert, amounting to 16 possible combinations of stimulus type and location. The two possible tactile-dominated stimuli comprised a phase that applied a 20-g force with a dowel or a brush to the palmar or plantar surface; the two possible proprioceptive-dominated stimuli applied extension or flexion to one of the limbs which included *move*, *hold*, and *return* phases. Dorsal column nuclei signals were simultaneously recorded from seven electrodes of a surface multi-electrode array (right insert). **(B)** Examples of 5 s of DCN signal recordings in response to stimulation of the left forelimb with each of the four stimulus types. Signals are color-coded according to their corresponding recording electrode shown in the right insert of **(A)**. The timing of stimulus phases is shown below each signal example for each stimulus type. Gray lines indicate the start and end of stimulus-on/-off periods. Abbreviations: DCN, dorsal column nuclei; e1, electrode 1; e2, electrode 2; e3, electrode 3; e4, electrode 4; e5, electrode 5; e6, electrode 6; e7, electrode, 7; SEM, standard error of the mean.

Rats were anesthetized with urethane (1.4g kg^−1^ i.p.). A tracheotomy was performed, and a breathing tube inserted to aid natural respiration. Rats were placed in a stereotaxic frame with head flexion at −20°; (Stoelting Instruments). The dorsal skin and muscles of the neck, and the dura and arachnoid mater were excised between the foramen magnum and the C1 vertebra to expose the dorsal surface of the brainstem. In most cases, the C1 vertebra was cut away with rongeurs to aid placement of an adapted surface multi-electrode array (sMEA; *Nucleus 22 Auditory Brainstem Implant*, Cochlear Limited). Details of the electrode dimensions are shown in the inset in Figure 1, and further details of the electrode can be found in Chelvanayagam et al. (2008). A flexible plastic rod held by a micromanipulator on the stereotaxic frame was lightly pressed onto the sMEA to hold it symmetrically over the brainstem midline.

### Stimulation and Recording

We applied two types each of tactile- and proprioception-dominated stimuli to the rat limbs amounting to 16 (4 stimuli × 4 limbs) possible mechanical stimulus conditions. The tactile-dominated stimuli were applied by pressing either a wooden dowel rod (diameter: 2.0 mm) or brush into the palmar/plantar pads of the paws. The brush was 25 mm in length, with diameter: 5.0 mm that tapered to a point over the last 10 mm. The rod and brush were fixed at the end of a flexible tube to deliver 20 g of force. The proprioception-dominated stimuli were applied to the rat by flexing or extending the rat’s limbs. The rats’ limbs were fixed to a dowel rod with ethyl cyanoacrylate instant adhesive (Loctite^®^, Henkel Adhesive Technologies) which enabled the experimenter to directly manipulate the limbs. Both flexion and extension involved the movement of the hip, knee, and ankle joints for the lower limbs, and the shoulder, elbow, and wrist joints in the forelimb. Therefore, the proprioceptive-dominated stimuli activated afferents across the entire limb, spanning three joints, and the associated muscles, in addition to the cutaneous afferents activated throughout the limbs by the movement. We do not describe the stimuli as exclusively tactile or proprioceptive, because the application of the tactile-dominated stimuli might also activate receptors in the joints and intrinsic muscles of the paw, and the proprioception-dominated stimuli undoubtedly activated tactile receptors associated with hair and skin throughout the limbs, in addition to the proprioceptive receptors in the skin, muscles, tendons, and joints. All stimuli were applied by the experimenter who was triggered by the timing of a metronome.

Stimuli were applied for 2.4 s with a rest period of 2.4 s. Tactile-dominated stimuli had a stimulus on/off pattern (4.8 s per trial), but proprioception-dominated stimuli also had a 2.4 s period which was used to return the limb to the resting state, where it remained in resting state for 2.4 s (7.2 s per trial). Stimuli were applied in 10 sets of 10 trials (100 stimuli per type), with 30 s rest between sets.

DCN electrical signals were acquired through seven electrodes of the sMEA and filtered (50 Hz notch filter; 10 kHz low-pass filter) through custom-built amplifiers. The signals were then digitally recorded (40 kHz sample rate) through a PowerLab 16/35 acquisition system and viewed in LabChart Pro software (Version 8.1.1, AD Instruments, Bella Vista, NSW, Australia). The seven electrodes of the sMEA are referred to from rostral to caudal as follows: left side electrodes: e1, e2; midline electrodes: e3, e4, e5; right side electrodes: e6, e7 (Figure 1A, right insert), and all seven electrodes combined are referred to as e1–7.

The large electrodes in this surface array were able to cover most, if not all, of the gracile nuclei, approximately three-quarters of the cuneate nuclei, and parts of the external cuneate nuclei. These electrodes allow acquisition of neural activity from large populations of neurons simultaneously and thereby permit finding the most salient and information-rich signal features that dominate somatosensory-evoked activity across most of the DCN, and how these features vary over time.

### Signal Processing and Feature Extraction

**Figure 2:**
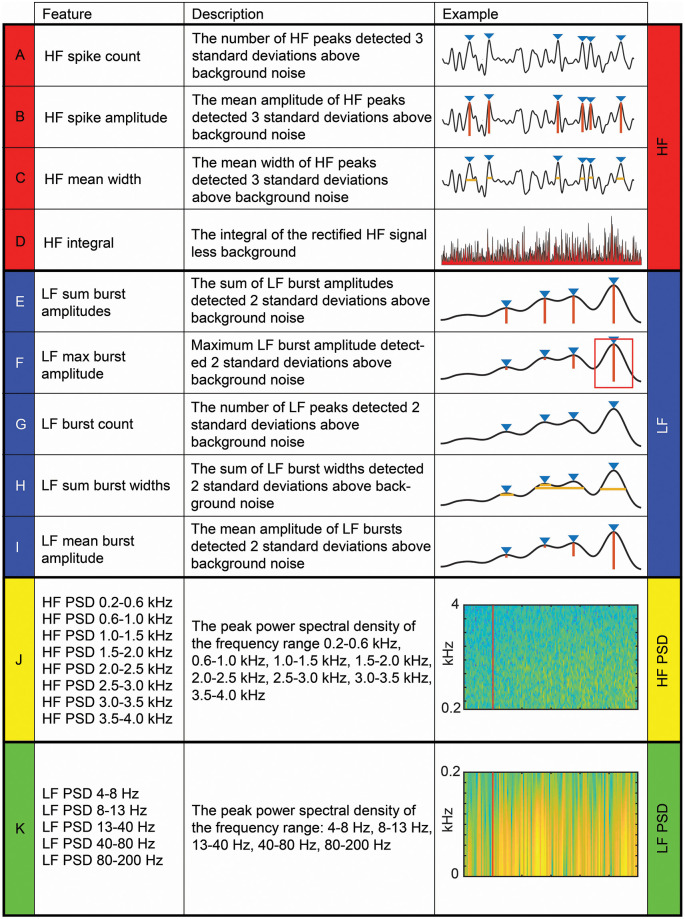
Four categories of 22 dorsal column nuclei signal features extracted for artificial neural network machine-learning. Descriptions and examples of individual features, extracted from dorsal column nuclei signals at various time windows, used to calculate feature-learnability (Loutit et al., 2019) are shown. Individual features were divided into four categories (color-coded): four HF **(A–D)**; five LF **(E–I)**; eight HF PSD **(J)**; and five LF PSD **(K)**. The red line in **(J)** and **(K)** indicates stimulus onset. Abbreviations: HF, high-frequency, LF, low-frequency; HF PSD, high-frequency power spectral density; LF PSD, low-frequency power spectral density.

Signal processing, feature extraction, and analysis were performed offline (MATLAB version R2018a, MathWorks). We used 22 DCN signal features as artificial neural network (ANN) inputs. The names, descriptions, and examples of feature extraction for all 22 features are shown in Figure 2. Features were categorized into LF (<200 Hz) and HF (>200 Hz) bands for analysis. Others have used a similar frequency (usually between 100 and 300 Hz) to separate LF activity that typically represents local field potentials, calcium spikes, and intrinsic neural membrane potentials, from HF activity that typically represents a single unit or multiunit spiking activity (Buzsáki et al., 2012). Four features were extracted from HF filtered (bandpass 0.55–3.3 kHz; 5-order Butterworth filter) signals (HF features; Figures 2A–D), and five from LF signals (LF features; Figures 2E–I), which were quantified from the HF signals after they had been rectified and low-pass filtered (<80 Hz; 5-order Butterworth filter). For all time-domain signals in which peak amplitudes were measured, we used the *prominence* output variable from the *findpeaks* MATLAB function. Thirteen features were extracted from frequency spectrograms (*spectrogram* MATLAB function) of DCN signals bandpass filtered between 4–5,000 Hz (8-order Butterworth). Eight of these features were quantified from the peak power spectral density of HF bands (HF PSD features): 200–600 Hz, 600–1,000 Hz, 1,000–1,500 Hz, 1,500–2,000 Hz, 2,000–2,500 Hz, 2,500–3,000 Hz, 3,000–3,500 Hz, 3,500–4,000 Hz (Figure 2J). The other five spectral features were quantified from the peak power spectral density of LF bands (LF PSD features): 4–8 Hz, 8–13 Hz, 13–40 Hz, 40–80 Hz, 80–200 Hz (Figure 2K). For all filtering, we used a zero-phase response filter: *filtfilt* function (MATLAB).

HF and LF features contain multiunit activity information, which in some cases outperforms spiking activity or local field potentials in offline decoding of motor tasks (Stark and Abeles, 2007) and has been used to control motor neural prostheses in tetraplegic patients (Flint et al., 2013; Bouton et al., 2016). The HF PSD features have been minimally investigated, but similar frequency band ranges have been used to decode motor signals in a neural prosthesis capable of effecting limb movement through neuromuscular stimulation (Bouton et al., 2016). The LF PSD features have been widely investigated in the motor cortex during reaching and grasping movements or used as decoding features for electrocorticographic or electroencephalographic neural prosthetic control (Wolpaw et al., 1991; Kostov and Polak, 2000; Leuthardt et al., 2004; Rickert et al., 2005; Flint et al., 2012; Chen et al., 2013; Marathe and Taylor, 2013; Bundy et al., 2016).

### Standardized Artificial Neural Network for Machine-Learning

Individual signal features extracted from each of the seven electrodes were paired to the stimuli that generated them, to create input/output pairs for machine-learning. All machine-learning experiments used a standardized ANN with a supervised learning classification algorithm (*patternnet* MATLAB function). The standardized ANN comprised 42 hidden neurons and 16 output neurons corresponding to the 16 possible stimuli (four stimulus types, presented to four different limbs). We selected the number of hidden neurons by increasing their number between 16 (the number of outputs) and 154 (the highest possible number of inputs from our feature set). For each number of hidden neurons, we determined the average feature-learnability with the minimum (7) and maximum (154) number of features included in the input set and found that 42 hidden neurons produced the highest average feature-learnability. Thus, only the input neurons were altered, depending on the number of input features required for each experiment. The ANN used gradient descent with momentum and the adaptive learning rate backpropagation training function *traingdx*. The hyperbolic tangent sigmoid function, *tansig*, was used in the hidden layer units, and a softmax transfer function (*softmax*) was used in the output layer. Both inputs and output targets were normalized such that all values fit between −1 and 1, as per the *patternnet* default setting. For training, cross-validation, and testing, the inputs were separated into training, validation, and test subsets, using the *patternnet* default settings.

### Feature-Learnability of Individual Features and Selection of Benchmark Input Feature Sets

Feature-learnability provides a stable measure of relevant information content provided by input features including a measure of the information content variability (Loutit et al., 2019). We used the Within Individual Animal (WIA) approach (Loutit et al., 2019) for all feature-learnability testing unless otherwise specified. In this approach, input/output pairs from 1,600 stimuli were generated for each animal. For each animal, ten repeated training (70%), validation (15%), and testing (15%) machine-learning cycles were performed under random initializing conditions. These ten confusion matrices were averaged to produce a single representative confusion matrix for each animal, then the representative confusion matrices for all animals were averaged. Feature-learnability was determined by the mean ± SEM derived from the diagonal of this matrix. To determine the feature-learnability of an individual feature, the inputs to the standardized ANN were restricted to the single input feature in question, resulting in a total of seven inputs (the signal feature extracted from each electrode).

To obtain a measure that represents the maximum possible information content contained in signal features, a benchmark feature set was determined that produces the highest feature-learnability outcomes. This feature-learnability benchmark facilitates comparisons of information content contained by individuals or subsets of input features. Individual features were extracted from all seven electrodes over the first 1,000 ms from stimulus onset, and feature-learnability determined. Individual features were then ranked from highest to lowest feature-learnability, prioritized by the largest means and smallest SEM (Loutit et al., 2019). Input feature selection was performed using an adapted sequential forward floating search algorithm (Whitney, 1971; Pudil et al., 1994). Our approach differed from a typical sequential forward floating search algorithm as we added four features (one from each feature category) to the input set per round of feature-learnability testing, rather than one feature. We also used a sequential backward search in each round to test whether removing features improved feature-learnability. The search was continued until the possible feature combinations presented by this method were exhausted and the maximum feature-learnability was determined.

A second benchmark was determined for features extracted over an optimal short-time window length (described below). The identical approach was used as for the 1,000 ms window benchmark, except that features were extracted from the shorter time course.

### Signal Feature Robustness Across Animals

To determine the generalisability of the features across animals we used the 1,000 ms window benchmark feature set to compare feature-learnability using a leave-one-out (LOO) approach. We randomly assigned input/output pairs from five animals into training (70%) and validation (30%) sets, and testing was performed on the remaining animal (100%). The LOO approach was applied such that each of the six animals was examined as the test data set once. For more details on these methods see Loutit et al. (2019).

### Feature-Learnability of Different Time Windows and Optimal Short-Time Window Selection

We sought to determine feature-learnability based on a shorter time-window for two purposes. Firstly, the features we investigated may be useful for decoding neural signals unrelated to DCN-specific signal features, such as neural signals from motor systems. Typical motor decoding algorithms use time windows ranging from 50–100 ms (Lebedev and Nicolelis, 2017), which trades classification accuracy for a feasible reduction in time required for real-time applications. Secondly, some peripheral somatosensory neural prostheses use stimulus patterns that mimic natural peripheral afferent firing properties, like variations in firing rate shown by fast and slowly-adapting afferents at the onset and offset of a stimulus. These biomimetic approaches have succeeded in eliciting more natural somatosensory percepts than simpler stimulus patterns that use only amplitude or frequency modulation to encode stimulus intensity (Valle et al., 2018; George et al., 2019). Therefore, determining feature-learnability over shorter time windows may help determine which features could inform the construction of artificial stimulus patterns during different stimulus events, such as contact onset, static holding, and contact offset. Extraction of features over longer time windows might mix those encoding onset, hold, and offset, and therefore be less informative to a decoding algorithm.

To determine an optimal short-time window that provides the highest feature-learnability in the shortest time, we tested windows between 20 ms to 150 ms with 10 ms increments, 250 ms, and 500 ms, in addition to the 1,000 ms windows described above. All windows started at the time of stimulus onset. We plotted feature-learnability against time windows for each feature and used the *findchangepts* MATLAB function to determine the most abrupt change in feature-learnability. We then averaged the changepoint values across all features and rounded to the nearest time window. A benchmark set of signal features was determined for the optimal short-time window (described above).

### Feature-Learnability Variations in Time During Stimulus Presentation

Once we established the optimal short-time window, we sought to use the short rolling window to extract features throughout stimulus presentation and determine how feature-learnability varied over time. To do this we extracted the 22 features from the optimal short-time window, sampled at 1,000 ms before the stimulus, then every 10 ms from 200 ms pre-stimulus to 4,000 ms post-stimulus. Finally, we tested feature-learnability for each of the features extracted from each of the 421 windows.

### Statistical Analysis

We used R (version 3.4.4; R Core Team, 2018) for all statistical analyses with the RStudio integrated development environment (version 1.1.442). For comparison of the feature-learnability benchmark within and across animals we used a linear model (LM; R *lm* function, *stats* package). For all other comparisons, we used a linear mixed-effects model (LMER; *lmer* function from the *lmerTest* package; Kuznetzova et al., 2019). Multiple comparisons were performed using estimated marginal means comparisons with Tukey *p*-value adjustments [R *emmeans* function, *emmeans* package (Lenth et al., 2019)]. We verified the model fitting by examining model residuals for normality. Where required, *log* or *logit* transformations (*car* package; Fox and Weisberg, 2019) were applied to the data before statistical modeling. All data are expressed as means ± SEM unless otherwise stated. Probabilities of *p* < 0.05 were deemed significant.

## Results

All raw data and extracted features from six animals are available in an open data repository (Loutit and Potas, 2020b).

### Tactile- and Proprioceptive-Dominated Stimuli Evoked Distinct Patterns of Neural Activity

Preliminary observations of the data revealed neural activity was greatest on midline electrodes and those ipsilateral to the site of stimulus. Tactile-dominated stimuli evoked activity that was greatest at stimulus onset and/or offset. Dowel stimuli generally evoked a short and sharp burst of neural activity that peaked within 10 ms of stimulus contact/removal, whereas the brush stimuli evoked a longer, ramped burst of neural activity that was comparatively delayed at onset/offset and to reach maximum (20–30 ms). In some cases, brush stimuli evoked two initial bursts at stimulus onset. Proprioception-dominated stimuli evoked more neural activity than tactile-evoked stimuli. In general, flexion resulted in greater neural activity compared to extension, however, the time to reach maximum neural activity was similar for both proprioception-dominated stimuli, which was at approximately the midpoint of the movement. Examples of filtered signals (0.55–3.3 kHz) acquired from seven electrodes in response to the four types of stimuli applied to the left forelimb are shown in Figure 1B. Features representing neural signals are shown in Figure 2.

### Feature-Learnability of Individual Features

**Figure 3:**
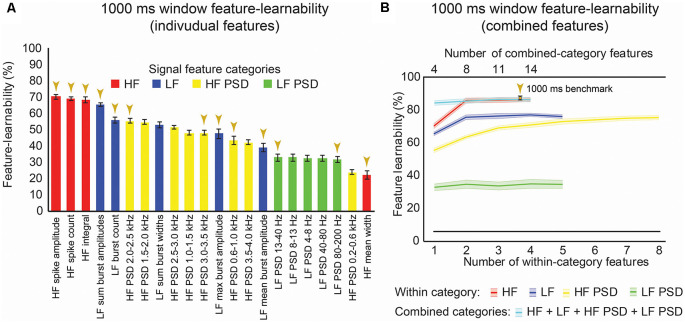
One-thousand milliseconds window benchmark determined from individual and combined signal features.** (A)** Feature-learnability was derived from dorsal column nuclei signal features extracted from 1,000 ms windows starting at the onset of each stimulus. Features are ordered in feature-learnability rank order (Loutit et al., 2019) from highest to lowest feature-learnability from left to right. Colors indicate the feature category that individual features belong (as described in Figure 2). Gold arrows indicate the 13 signal features that comprise the 1,000 ms window benchmark configuration also indicated in panel **(B)**. **(B)** Feature-learnability was determined after consecutively adding individual features from the same category (within-category features) that improved classification accuracy. The number of individual features included in within-category combinations is indicated by the bottom x-axis; curves are color-coded according to the within-category features. Feature-learnability was also determined by combining the within-category features from all four categories (combined-category features, plotted in cyan). The total number of individual features included in the combined-category feature combinations is indicated by the top x-axis; the gold arrow indicates the 1,000 ms window benchmark configuration for all subsequent comparisons. The black line indicates a chance level of classification (6.25%). Feature-learnability data expressed as mean ± SEM. Abbreviations: HF, high-frequency, LF, low-frequency; HF PSD, high-frequency power spectral density; LF PSD, low-frequency power spectral density. See Figure 2 for feature name descriptions.

We sought to rank individual features for their capacity to be informative of stimulus type and location. Figure 3A shows the rank order of feature-learnability for all 22 features extracted from the first 1,000 ms following the stimulus onset across all seven electrodes. All 22 features performed significantly greater than chance levels of 6.25%. Feature-learnability ranking clustered into three groups: (1) highest-performing features, comprising three of the four HF and one of the five LF features; (2) middle performing features, comprising the remaining LF and all but one of the HF PSD features; and (3) the lowest-performing features, comprising mainly LF PSD feature and the remaining HF and HF PSD features, all of which still performed three times greater than chance levels. These findings indicate that time-domain HF features are the most informative for determining a combination of stimulus location and type.

### Establishing a 1,000 ms Window Feature-Learnability Benchmark

We previously demonstrated that combinations of LF and HF signal features improve machine-learning outcomes (Loutit et al., 2019). We, therefore, sought to determine a combination of input features that produced the highest feature-learnability and to establish a benchmark for subsequent comparisons. Combinations derived from the best performing pair of features from within the HF, LF, and HF PSD categories resulted in significant feature-learnability improvements, compared to single features (*p* ≤ 0.01, LMER, Tukey), but tended to plateau after adding the second feature (Figure 3B). The best performing pair of frequency-domain features in the LF PSD category was not better than the best performing single LF PSD feature (*p* = 1.0, LMER, Tukey). The highest-ranked learnability from within-category feature combinations was achieved by combining all four HF features (86.5%). However, this HF 4-feature combination was not significantly greater than the 2 or 3-feature HF combinations (*p* = 1.0, LMER, Tukey), all of which also significantly out-performed the best individual HF feature and all other within-category combinations (*p* ≤ 0.0003, LMER, Tukey; Figure 3B).

To determine if combinations of features from different categories improved feature-learnability, we combined the highest-ranked feature from each category (i.e., a combination with 4 features). This combination yielded significantly greater feature-learnability than all LF, LF PSD, and HF PSD highest-ranked feature combinations (*p* ≤ 0.0063, LMER, Tukey). Compared to the HF feature category, the 4-feature across-category combination was only significantly greater than the highest-ranked single HF feature (*p* < 0.0001, LMER, Tukey), but was not significantly different when additional HF features were added (*p* = 1.0, LMER, Tukey; Figure 3B).

**Figure 4:**
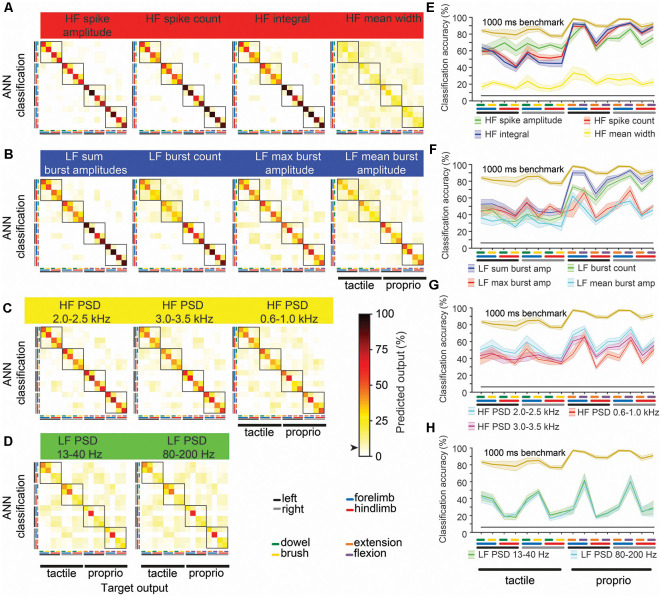
Machine-learning outcomes for individual and combinations of 1,000 ms window benchmark features. Panels **(A–D)** shown are confusion matrices (mean of six animals) of machine-learning outcomes from 13 features comprising the benchmark feature set (Figure 3A) grouped by their four categories (Figure 2). Features in the left column were the first four features combined (blue curve, Figure 3B), and each confusion matrix to the right shows successive additions to the feature set. The black arrow on the color bar below confusion matrices indicates a chance level (6.25%). **(E–H)** The diagonal of each confusion matrix is plotted in the line graphs, with features in **(A–D)** corresponding to line graphs **(E–H)**, respectively. Dark lines in **(E–H)** show means of six animals and the corresponding pale bars indicate ± SEM; colors that are arbitrarily chosen. Superimposed is the benchmark feature-learnability (gold) for comparison; black straight lines indicate classification chance level (6.25%). See Figure 2 for feature descriptions.

To find the combination with the highest-ranked feature-learnability, we sequentially added the next best features in groups of four (i.e., the next best individual feature from each of the four categories). The highest-ranked feature-learnability achieved was 87.2 ± 1.3% with 13 features which we defined as our 1,000 ms window feature-learnability benchmark for subsequent comparisons. Despite improved feature-learnability with 13 features compared to 4, 8, or 11 features, there were no significant differences between feature-learnability outcomes of any of the across-category combined feature sets (p ≥ 0.66, LMER, Tukey; Figure 3B). The individual features that contribute to the 1,000 ms benchmark combination are indicated in Figure 3A, and their confusion matrices are shown in Figures 4A–D.

In summary, time-domain HF features resulted in the highest feature-learnability rankings, and although the benchmark feature set included 13 features from all four categories, benchmark feature-learnability was not significantly higher than the combination of two HF features (*HF spike amplitude* and *HF spike count*).

### How Well do Individual Features Predict Different Mechanical Somatosensory Stimuli?

To determine what information the 1,000 ms benchmark input features contribute to feature-learnability, we plotted confusion matrices for all 13 features (Figure 4). Correct predictions (i.e., the diagonal) of each matrix are replotted with their SEM in the right panels of Figures 4E–H to facilitate performance comparisons of each feature and to provide a measure of variability among animals.

The HF feature category (Figure 4A) significantly outperformed all other categories (*p* ≤ 0.001, LMER, Tukey). Proprioception-dominated stimuli were significantly better classified than tactile-dominated stimuli by all categories (*p* < 0.0001, LMER, Tukey), except the LF PSD category (Figure 4H, *p* = 0.74, LMER, Tukey). The forelimbs were significantly better classified than hindlimbs across all categories (*p* ≤ 0.004, LMER, Tukey), and for both tactile- and proprioceptive-dominated stimuli (*p* < 0.0001, LMER, Tukey). *HF spike amplitude* was the best predictor of tactile-dominated stimuli and significantly outperformed all other features (*p* ≤ 0.0026, LMER, Tukey; Figures 4E–H). *HF integral* was the best predictor of proprioceptive-dominated stimuli and significantly outperformed most other features (*p* ≤ 0.0019, LMER, Tukey), except *HF spike count* and *LF sum burst amplitudes* (*p* 1.0, LMER, Tukey; Figures 4E–H). Interestingly, the *LF burst count* predicted proprioceptive-dominated stimuli significantly better when evoked from right limbs compared to left limbs (*p* = 0.035, LMER, Tukey; Figure 4F).

### HF Feature Quantification

To investigate how *HF spike amplitude* predicted tactile stimuli significantly better than all other features, including the next highest performing feature *HF spike count*, we quantified *HF spike amplitude* and compared this to *HF spike count* acquired from each electrode and animal. We previously demonstrated that a feature’s learnability is correlated to the number of instances different stimuli evoke significantly different magnitudes of that feature (Loutit et al., 2019). We, therefore, quantified the two features from anatomically relevant electrodes, i.e., features acquired from stimuli applied to left forelimbs were quantified from left electrodes (e1 and e2), right forelimbs from right electrodes (e6 and e7), and hindlimbs from midline electrodes (e3, e4, e5).

Although the effect size was small, *HF spike amplitude* evoked by dowel stimuli (30.2 ± 1.2 μV) were significantly higher than brush stimuli (29.7 ± 1.0 μV; *p* = 1.3e-4, paired *t*-test), as were *HF spike counts* (dowel, 41.6 ± 2.5 events; brush, 40.0 ± 1.9 events; *p* = 0.041, paired *t*-test). *HF spike amplitudes* evoked by flexion (32.4 ± 1.2 μV) were significantly higher than when evoked by extension (31.1 ± 1.2 μV; *p* = 2.1e-12, paired *t*-test), as were *HF spike counts* (flexion, 129.1 ± 8.2 events; extension 87.9 ± 5.9 events; *p* = 3.3e-14). Moreover, *HF spike amplitude* of proprioception-dominated stimuli (31.7 ± 0.5 μV) was significantly higher than when evoked from tactile-dominated stimuli (29.8 ± 0.5 μV; *p* = 0.013, Student’s *t*-test), and proprioception-evoked *HF spike counts* (108.5 ± 5.7 events) were more than 2.5 times larger than when tactile-evoked (40.8 ± 2.0 events; *p* = 8.4e-24, Student’s *t*-test).

In summary, despite small effect sizes, the difference in the *HF spike amplitude* evoked from dowel vs. brush stimuli resulted in a much smaller probability value compared to *HF spike counts*, which may account for the improved tactile classification outcome from *HF spike amplitude*. Dowel, flexion, and proprioception-dominated stimuli evoke higher *HF spike amplitudes* and *HF spike counts* than a brush, extension, and tactile-dominated stimuli, respectively.

### Signal Feature Robustness Across Animals

**Figure 5:**
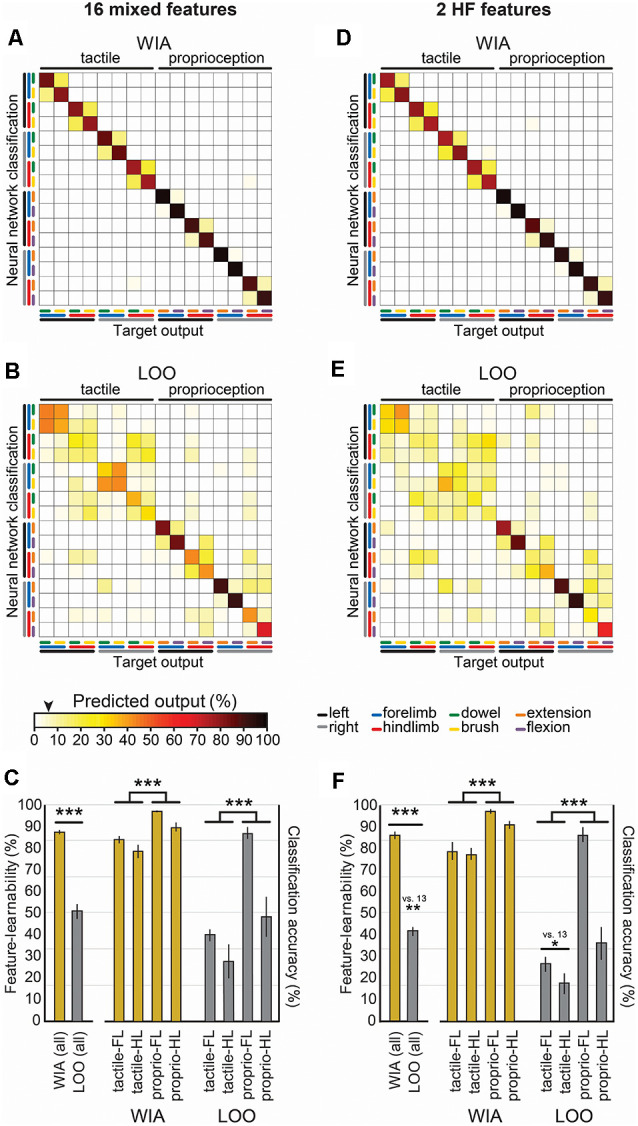
Feature robustness across animals. Confusion matrix means are shown for WIA and LOO machine-learning models. These models use identical neural network architecture extracted from 1,000 ms post-stimulus onset, but differ by their training, validation, and testing data partitioning approaches. **(A)** The confusion matrix shows the mean values derived using the WIA approach for all six individual animals using the 13 mixed features that constitute the 1,000 ms feature-learnability benchmark (Figure 3). The WIA approach partitions training, validation, and testing datasets from within individual animals (Loutit et al., 2019), thus machine-learning models are optimized for each animal. **(B)** The confusion matrix shows the mean values derived using the LOO approach for all six individual animals on the same input data set as **(A)**. The LOO approach trains machine-learning models from all other (*n* = 5) animals and tests on the remaining animal, and thereby quantifies feature-learnability for each animal against the background of all others. **(C)** A comparison of the feature-learnability (left bars) for the WIA (gold bars) and LOO (gray bars) approaches are shown [derived from the means ± SEM of the diagonals in **(A,B)** respectively]. The large significant reduction of feature-learnability indicates that a large portion of information encoded in the signal features was unique to individual animals, but that a significant portion also generalizes across animals. The right bars show classification accuracy for stimulus classes across limbs (derived from means ± SEM calculated from the six confusion matrices from all animals) for WIA (gold) and LOO (gray) approaches. LOO output demonstrates the 13 signal features are highly informative for all animals for the forelimb proprioception class, but significantly less informative for all hindlimb and tactile classes. **(D)** Same analysis as **(A)**, but WIA inputs restricted to the two best HF features (*HF spike amplitude* and *HF spike count*; Figure 3). **(E)** Same data set as **(D)** using the LOO approach. **(F)** Identical analysis as per **(C)** but for the two best features **(D–E)**. The LOO approach output for these two best features demonstrates a similar pattern shown for the 13 best features shown in **(C)**, indicating that *HF spike amplitude* and *HF spike count* generalize across animals for decoding forelimb proprioception-dominated stimuli. The black arrow on the color bar below confusion matrices indicates a chance level (6.25%). Abbreviations: WIA, within individual animals; LOO, leave-one-out; LF, forelimb; HF, hindlimb. *p < 0.05, **p < 0.01, ***p < 0.001.

How generalizable are signal features of the 1,000 ms benchmark across different animals? To answer this question, we used the LOO approach to measure how features extracted from an individual animal perform when presented to a neural network trained by the same features derived from the remaining cohort of animals. This approach measures how features from one animal generalize to all other animals (Loutit et al., 2019). Compared to the WIA approach derived from neural networks optimized for individual animals using 13 features over 1,000 ms (Figure 5A), feature-learnability determined by the LOO approach (Figure 5B) was significantly reduced, by almost half to that of the WIA approach (Figure 5C; *p* < 2.2e-16, LMER). More than 50% of the reduction in feature-learnability derived by the LOO approach was accounted for by greater confusion errors associated with tactile-dominated stimuli, specifically, that dowel and brush stimuli were generally poorly discriminated, which were exacerbated by left/right errors for the hindlimb (Figures 5B,C). Compared to the WIA approach, proprioception-dominated stimuli from the LOO approach demonstrated an insignificant 11% reduction in classification accuracy at the forelimb (*p* = 0.25, LMER, Tukey), but a 43% reduction associated with the hindlimb (*p* < 0.0001, LMER, Tukey; Figure 5C). These results indicated that the 13 features contain information that is highly generalizable among animals for proprioceptive-dominated stimuli of the forelimb, but much less so for hindlimb proprioception and forelimb and hindlimb tactile-dominated stimuli.

Combining the two highest-ranked HF features (Figure 3) resulted in WIA feature-learnability (85.6 ± 1.2; Figure 5D) not significantly different from the combination of 13 features (87.2 ± 1.3; Figure 5A, *p* = 0.13, LMER). To determine how these two features alone generalize across animals, the LOO approach was applied (Figures 5E,F). The LOO approach restricted to the two highest-ranked HF features revealed an almost identical pattern as the 13 features data set, but with the addition of forelimb/hindlimb confusion for tactile-dominated stimuli (Figures 5E,F). Compared to the 13-feature combination, LOO feature-learnability of the 2-feature combination was overall significantly reduced (*p* = 0.002, LMER; Figure 5F), which resulted because of reduced tactile stimulus classification accuracy that was generalized across both hind- and forelimbs (*p* = 0.025, Tukey; Figures 5E,F). Interestingly, there was no significant reduction in proprioception performance in the LOO approach between the 13- and 2-feature sets (*p* = 0.37, LMER, Tukey).

These results indicate that the two HF features extracted over 1,000 ms can provide almost all of the information provided by the larger 13-feature set for forelimb proprioceptive-dominated stimulation, but the other 11 features contribute some additional information, common across all animals, which is important to discriminate hindlimb proprioceptive-stimuli as well as fore-and hindlimb tactile-dominated stimuli.

### Learnability of Features Extracted Over Different Time Windows

**Figure 6:**
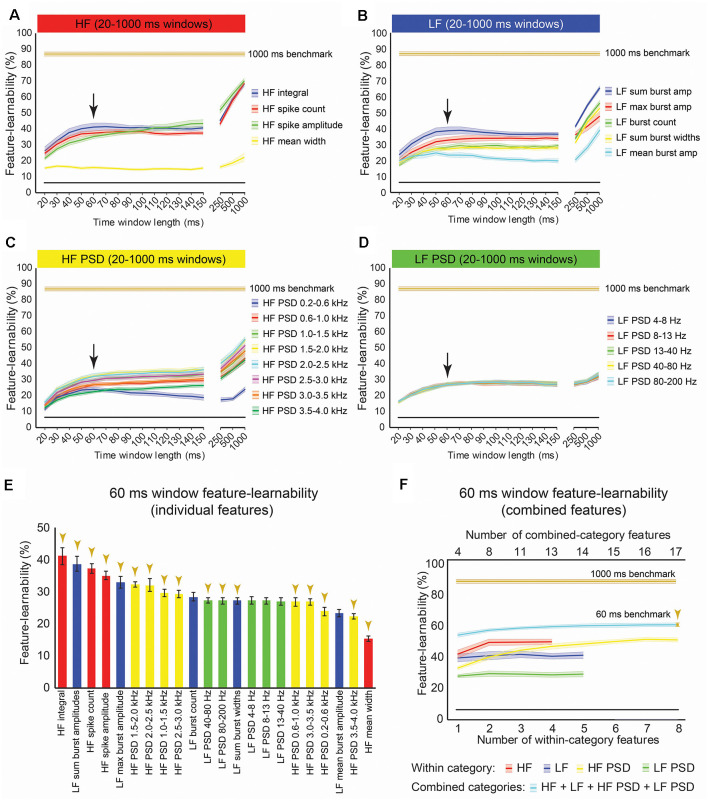
Benchmark signal features for an optimal short-time window. **(A–D)** Feature-learnability of features extracted from time windows of varying lengths from 20 ms to 1,000 ms is shown in their HF **(A)**, LF **(B)**, HF PSD **(C)**, and LF PSD **(D)** signal feature categories. Colored lines and shading indicate the feature-learnability (mean ± SEM) for each signal feature (colors arbitrarily chosen). Gold lines indicate the 1,000 ms window benchmark feature-learnability ± SEM (see Figure 4D) for comparison; black straight lines indicate classification chance level (6.25%). Arrows indicate the 60 ms window as the optimized duration across all features where the return of feature-learnability diminishes for increasing time windows. **(E)** Feature-learnability ranking of individual signal features is shown for the optimized short-time window (i.e., 60 ms). Gold arrows indicate the 13 signal features that comprise the benchmark configuration. **(F)** Benchmark signal features were determined for the optimized short-time window using the identical algorithm applied to determine the 1,000 ms window benchmark feature set shown in Figure 3A; blue curve shows feature-learnability from combining across categories; gold arrow indicates benchmark configuration of 17 features (indicated in E). For comparison, the 1,000 ms window benchmark feature-learnability is shown (gold curve); the black straight lines indicate classification chance level (6.25%). See Figure 2 for feature descriptions and abbreviations.

Features extracted over a 1,000 ms period have little practical value for neural prosthetic feedback control. A DCN-targeted neural prosthetic device would need to provide stimulus features over shorter time windows to enable rapid updating of limb sensory status. To determine how learnable DCN signal features are over shorter periods, feature-learnability was calculated for each signal feature extracted over time windows ranging from 20–500 ms starting from stimulus onset (Figures 6A–D). A window of 60 ms was determined as the time of most abrupt change in feature-learnability across all features (see arrows, Figures 6A–D) before reaching a plateau, where feature-learnability improved slightly, or not at all until much larger time windows (≥250 ms). Feature-learnability rank order derived from features extracted from 60 ms windows (Figure 6E) was altered compared to 1,000 ms windows (Figure 3A). Notably, *HF integral* out-ranked *HF spike amplitude* (the best feature from 1,000 ms windows), although these features were not significantly different (*p* = 0.052, LMER, Tukey; Figure 6E), and the second-ranked feature was *LF sum burst amplitudes* (ranked 4^th^ from 1,000 ms windows). *HF’s mean width* remained the lowest-ranked feature.

Combining the best two features (*HF integral* + *LF sum burst amplitude*) did not significantly improve feature-learnability (41.8 ± 2.2) compared to *HF integral* alone (41.0 ± 2.5, *p* = 0.09, paired *t*-test). A combination of four signal features that included the highest-ranked feature from each category extracted from 60 ms, significantly improved feature-learnability compared to most within-category combinations, except for the combination of two or more HF features (*p* ≥ 0.76) and five or more HF PSD features (*p* ≥ 0.35, LMER, Tukey; Figure 6F). Also noteworthy was that feature-learnability from combining *HF integral* and *HF spike amplitude* was significantly greater than all other within-category feature combinations, except for three or more combined HF PSD features, all of which were not significantly different (*p* ≥ 0.6, LMER, Tukey). The 60 ms benchmark feature set, determined by the continual addition of the next best features from each category (where possible), resulted in a feature-learnability outcome of 59.4% from 17 features (gold arrows, Figures 6E,F).

### Temporal Profiles of Feature-Learnability During Mechanical Stimuli

**Figure 7:**
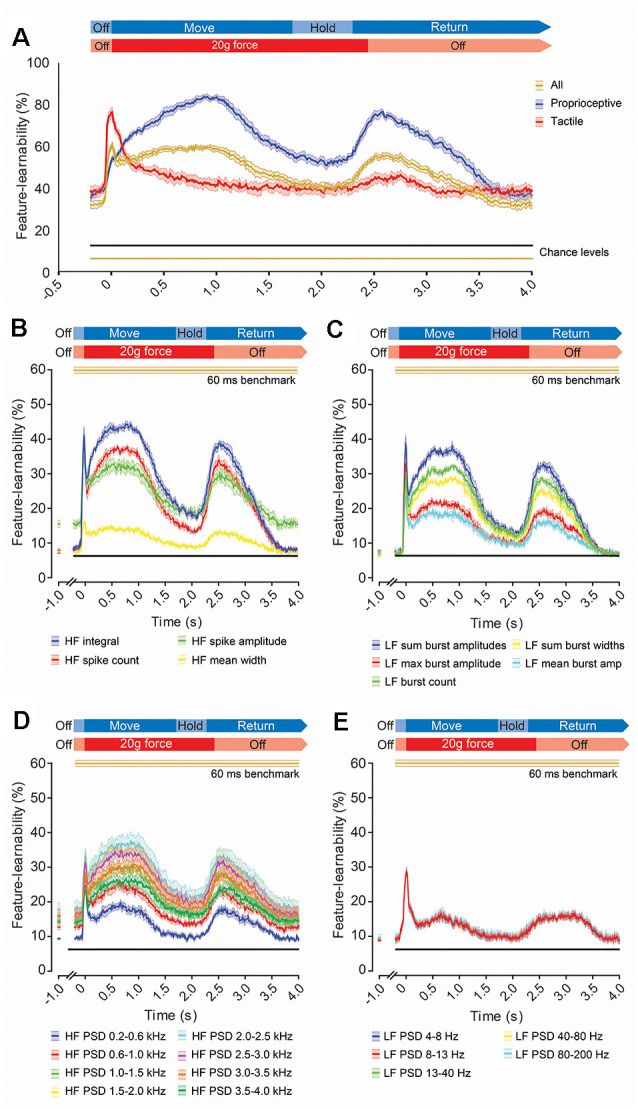
Evolution of feature-learnability from 60 ms windows during natural mechanical stimuli.** (A)** Feature-learnability time-series determined by extracting features from a 60 ms sliding window advanced every 10 ms are shown for the 60 ms benchmark feature set of 17 features (Figure 6E) for all stimuli (gold trace), as well as modified neural networks restricted to proprioceptive- or tactile-dominated inputs/outputs only (indicated by blue and red traces respectively). Expected feature-learnability by chance is indicated for all (gold) and proprioceptive- and tactile-dominated (black) stimuli. **(B–E)** Feature-learnability time-series at 1,000 ms pre-stimulus and from 200 ms pre-stimulus to 4,000 ms post-stimulus are shown in their HF **(B)**, LF **(C)**, HF PSD **(D)**, and LF PSD **(E)** signal feature categories. Colored lines and shading indicate the feature-learnability (mean ± SEM) for each signal feature (colors arbitrarily chosen). Gold lines indicate the 60 ms window benchmark feature-learnability ± SEM (Figure 6F) for comparison; black straight lines indicate classification chance level (6.25%). See Figure 2 for feature descriptions and abbreviations.

To measure how informative signal features, extracted over a short period, are throughout stimulus presentation, we determined a feature-learnability time-series by extracting DCN signal features from 60 ms rolling windows (Figure 7). We started by examining the feature-learnability temporal profile for the 60 ms benchmark 17-features set (indicated by gold arrows, Figure 6E). This produced a complex waveform (gold trace, Figure 7A) with three distinct peaks: the 1st is an abrupt, relatively sharp peak coinciding with the stimulus onset; the 2nd and 3rd peaks were broadened and peaked at the approximate midpoint of the stimuli and beginning of the stimulus-off/return phase, respectively. To establish which stimuli contributed to these peaks, neural networks were restricted to input/output data sets of tactile- or proprioception-dominated stimuli (Figure 7A, red and blue traces, respectively). This revealed that the 1st peak arose from tactile-dominated stimuli, the 2nd peak arose from proprioceptive-dominated stimuli, whereas the 3rd peak arose from both tactile- and proprioceptive-dominated stimuli. Another interesting observation was that during the stimulus-off/return phase, feature-learnability was significantly elevated from chance levels, indicating that the input features during this period were informing the neural network of some information about the stimulation being performed.

To determine how individual features contribute to feature-learnability over this time course, we examined feature-learnability time profiles for each of the 60 ms benchmark features (Figures 7B–D). For all features, the 3rd peak coincided with the commencement of the off-stimulus period and their amplitudes never exceeded their respective 1st or 2nd peaks. Of the four time-domain HF features, the maximum peak for three of these coincided with the 1st (tactile) peak, whereas the *HF integral* maximum fell on the 2nd (proprioceptive) peak (Figure 7B). Feature-learnability returned to near-chance levels during the stimulus-off/return period for all HF features except *HF spike amplitude*, which remained significantly elevated. Of the five time-domain LF features, the maximum peak for two of these coincided with the 1st peak (*LF max burst amplitude* and *LF mean burst amplitude*), whereas the other three features had similar 1st and 2nd peak magnitudes (Figure 7C). Feature-learnability returned to chance levels during the stimulus-off/return period for all LF features.

Of the eight HF PSD features, the maximum peak coincided with the 1st peak for two features derived from frequencies between 0.2–1.0 kHz, whereas maximum feature-learnability coincided with the 2nd peak for features derived from frequencies between 1.5–4.0 kHz (Figure 7D). All HF PSD features remained elevated above chance levels during the stimulus-off period, with frequencies closer to the 2 kHz range demonstrating greater feature-learnability during the stimulus-off/return period. For all five LF PSD features, the maximum peak coincided with the 1st peak, and all remained slightly elevated above chance levels during the stimulus-off/return period (Figure 7E).

**Figure 8:**
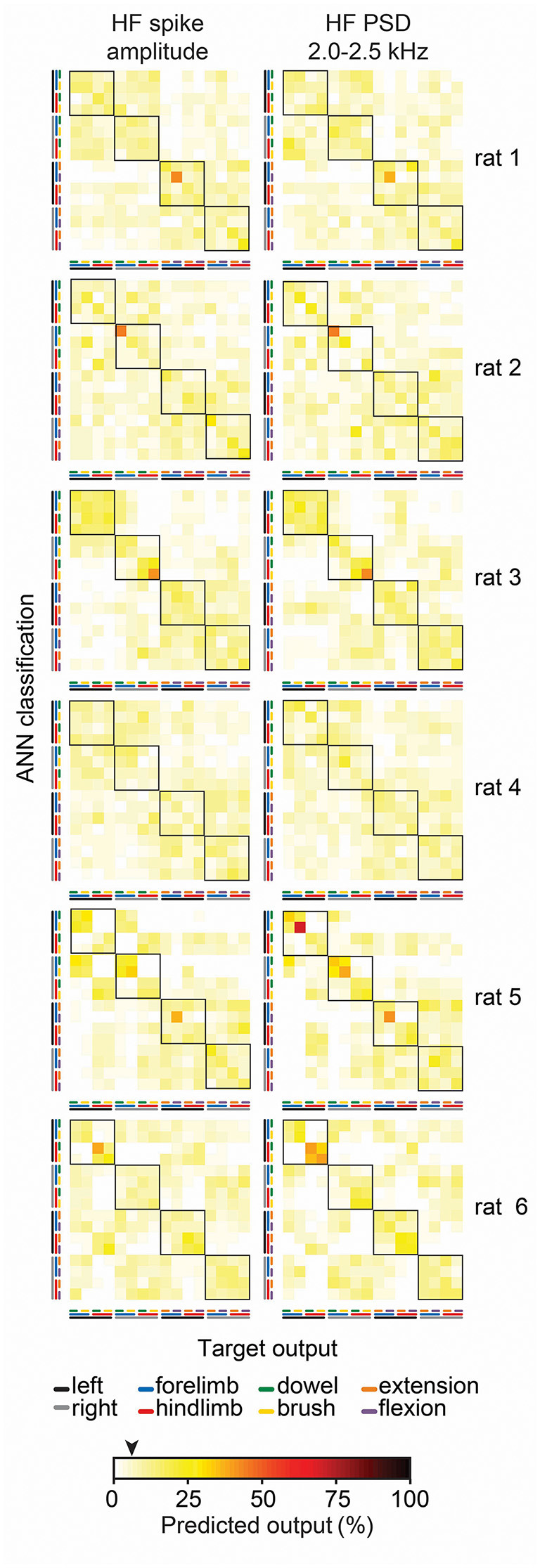
Individual animal machine learning outcomes for 60 ms window at 1 s pre-stimulus. Each confusion matrix shows the machine learning outcomes for individual animals (rows) for two features (columns) which demonstrated feature-learnability significantly above chance levels at 1 s pre-stimulus. Note the similarity within animals, but lack thereof across animals. The black arrow on the color bar below confusion matrices indicates a chance level (6.25%).

To gain insight into why feature-learnability remained above chance levels during the stimulus-off/return period, we inspected confusion matrices from all animals for features associated with above-chance performance at 1-s pre-stimulus. Figure 8 shows examples of two features (*HF spike amplitude* and *HF PSD 2.0–2.5 kHz*), which demonstrates that increased feature-learnability 1 s before stimulus arose for different reasons across different animals, but similar reasons within animals. One common theme was that the correct limb was identified for the two stimulus categories. In all but one animal, one or more outputs were correctly classified with >35% accuracy. To investigate if there were learning differences at the beginning vs the end of trials, we then divided data sets into thirds for: (i) each of the 10 trials; and (ii) for the 100 sequential stimulus presentations, to see if repeated stimuli within and across the trials contributed to improved outcomes. We found no difference in feature-learnability between the first-third and last-third of stimulus presentation within-trial sets, or across all 100 trials, indicating that there were no changes to feature-learnability as a result of repeating trials.

In summary, for 60 ms rolling windows some features performed better over periods rich in tactile-stimulus information and others over the proprioception-rich periods. PSD features derived from <1kHz, contributed more during tactile-rich periods, whereas that >1.5kHz contributed more toward proprioception-rich periods, while feature-learnability during the stimulus-off/return period was greatest for PSD features around 2 kHz and reduced for lower and higher frequencies. The reasons for above-chance performance during the stimulus-off/return period appears non-generalizable because it was different for each animal.

## Discussion

Our study used feature-learnability to reveal several neural signal features that facilitate excellent decoding accuracy for mechanically-evoked tactile- and proprioception-dominated stimuli over a range of frequencies (4–4,000 Hz). We demonstrated that individual features extracted over 1,000 ms of data from the HF category generally outperformed those from other categories and that only two HF features from somatosensory DCN-signals were required to achieve the 1,000 ms feature-learnability benchmark. With a shorter time-window of 60 ms—one that is compatible with real-time applications—decoding accuracy and robustness of signal features were greatly improved by adding relevant and diverse features, and reasonable classification accuracy was achieved despite sampling from electrodes with poor spatial resolution. We found proprioception-dominated stimuli were more accurately classified than tactile-dominated stimuli, and stimuli presented to the forelimbs were predicted better than hindlimbs. Our study established time courses that track how information content, for each feature, varies as a function of the mechanical stimulation phases. We discuss these findings below concerning the underlying DCN physiology after the following methodological considerations.

The sMEA used to capture surface potentials had relatively poor spatial resolution and a low number of electrodes. Others have shown that recording with higher electrode densities will permit greater classification accuracy (Mehring et al., 2003; Bansal et al., 2011; Wong et al., 2016). Despite the low resolution, however, we found several features that reproducibly represent encoded somatosensory signals in the DCN leading to high classification accuracy. This suggests that many of the investigated features accurately predict DCN population activity, without the need for precise spatial resolution, and are therefore common or salient DCN activity features that may be used to inform the creation of biomimetic stimulus patterns (Saal and Bensmaia, 2015; George et al., 2019; Loutit and Potas, 2020a). For example, some LF features showed high feature-learnability at stimulus onset, but low feature-learnability during later phases of the stimulus. If LF features represent large populations of neural activity (see “LF Features” section) then stimuli could be constructed to generate bursts of activity in large populations of neurons to signify the onset of a stimulus, but other features (likely HF features) may inform how to activate specific neurons during the later phases of the stimuli when many neurons have adapted to a stimulus. We, therefore, suggest that future DCN stimulation approaches would benefit from strategically placed, high-density electrode arrays that can activate specific neural populations within the DCN.

We described our stimuli as either tactile- or proprioception-dominated. While these stimuli target most of the intended afferents under investigation, we must acknowledge the diversity of afferents being recruited by our natural stimuli. For example, the 20 g force applied by the tactile-dominated stimuli to the palmar/plantar surfaces are likely to have also moved wrist/ankle and finger/toe joints, while the proprioception-dominated stimuli are likely to have activated hair and skin afferents around joints, on resting surfaces of the limb, and the skin where the actuator was attached that moved the limbs. These stimuli, therefore, do not activate tactile and proprioception afferents in isolation; however, the time courses of neural responses were characteristic of tactile and proprioceptive afferent firing, indicating that most intended afferents were appropriately activated. It is also worth noting that any natural stimulus will activate a mix of afferent populations and therefore activation of a pure afferent type would be a rare occurrence in nature.

### Prediction of Somatosensory Stimuli From Dorsal Column Nuclei Signals

Across all individual features and our benchmark feature set, proprioception-dominated stimuli were generally predicted better than tactile-dominated stimuli, and forelimbs were predicted better than hindlimbs. More accurate proprioception classification compared to tactile is likely to have resulted because there was a clear significant separation in the neural activity evoked by flexion and extension (indicated by the *HF spike count* feature alone), and both these proprioceptive stimuli evoked activity that was significantly different to both tactile stimuli, whereas, the significant difference and effect size was much less between the two tactile stimuli. Greater proprioception evoked activity may have resulted from activating more receptors and/or evoking more action potentials from each activated receptor, as described above. Furthermore, tactile stimuli evoked most activity at stimulus onset, then appeared to quickly adapt (Figures 1B, 7A), whereas moving the limb evoked high activity levels throughout the entire duration of both proprioceptive stimuli, thus the potentially higher number of proprioceptors were also active for a longer duration.

Forelimbs may have been predicted better than hindlimbs because most hindlimb activities were acquired by the same midline electrodes which spanned across the two gracile nuclei on both sides, whereas each cuneate nucleus on either side had its electrode, thereby facilitating better spatial discrimination. Some of the hindlimb errors resulted from confusing tactile- and proprioceptive-dominated stimuli of the same limb (Figures 4A–D, 5B,E). Most hindlimb proprioceptive afferents either project onto DCN neurons in the ventral gracile nuclei or to nuclei X and Z, which are rostral nuclei that form part of the DCN-complex (for a comprehensive review see Loutit et al., 2020). Although both hindlimbs tactile and proprioceptive afferents project to the DCN, the proprioceptive DCN regions may be too deep to acquire HF features, while nuclei X and Z were not covered by the placement of our electrode array. Thus, prediction of hindlimb stimuli may have relied mostly on tactile information to discriminate between the tactile and proprioceptive-dominated stimuli and were confined to midline electrodes, thereby reducing overall lower hindlimb prediction accuracy. Meanwhile, the external cuneate nuclei, part of the DCN-complex that exclusively receives forelimb proprioceptive afferents (Loutit et al., 2020), were located partially under the lateral electrodes. Thus, tactile evoked forelimb activity was mainly acquired from both lateral and midline electrodes, whereas proprioceptive evoked forelimb activity was acquired mainly from lateral electrodes. The segregation of proprioceptive information across separate electrodes for forelimbs, but not hindlimb-evoked activity, is likely to explain the reduced feature-learnability derived from hindlimb stimuli.

### Feature-Learnability Within and Across Animals

To determine if benchmark features are generally useful for robust decoding of mechanical stimuli in different animals and not unique to individual animals, we quantified feature-learnability using the LOO approach. This approach trains the machine-learning algorithm on features extracted from all other animals and tests on the remaining animal. We previously established that comparing feature-learnability under LOO and WIA conditions provide insight into how well, or poorly, DCN-signal extracted features generalize across animals (Loutit et al., 2019).

Forelimb flexion and extension were minimally perturbed under LOO conditions, indicating that 1,000 ms benchmark features were highly relevant across all animals for forelimb proprioception stimuli. However, LOO conditions reduced feature-learnability by 37%, which was mostly due to a more than 50% reduction in the ability to predict tactile stimuli and a 40% reduction in the ability to predict hindlimb proprioception-dominated stimuli. This indicates that a significant portion of the information contained within benchmark features are no longer relevant, or as generalizable, for tactile- and hindlimb-presented stimuli across the different animals.

Hindlimb stimuli classification errors (both tactile- and proprioception-dominated) mainly resulted from confusing the side of the body that a stimulus was presented. Signal asymmetry across the DCN surface, as we have previously shown for hindlimb stimulated nerves (Loutit et al., 2017, 2019), could lead to larger signal variations in left and right limb-derived activity, across the different animals we observed in the present study.

### High-Frequency Features

*HF spike amplitude* was the best performing feature for the 1,000 ms windows, and fourth in the 60 ms window, yet, to our knowledge, this signal feature has not been previously used for neural decoding. The *HF spike amplitude* feature resulted in fewer errors when classifying brush and dowel stimuli of the same limb, compared to all other features (Figure 4), which implies that the machine-learning algorithm detected greater contrast in this feature’s magnitude under the two tactile stimulus conditions compared to other features. We observed that at stimulus onset, dowel stimuli produced more precisely timed bursts of activity than brush stimuli. This may have facilitated the improved dowel/brush classification from the *HF spike amplitude* feature because spikes from multiple neurons or afferent fibers arriving at an electrode simultaneously (i.e., responses evoked by dowel stimuli) will summate, and therefore show higher average peak amplitudes than single spikes that have less temporal overlap (i.e., brush stimulus-evoked responses). This explanation is supported by the observed significantly greater *HF spike amplitude* evoked by a dowel, compared to brush stimuli.

The combination of *HF spike amplitude* and *HF spike count* features, extracted over 1,000 ms, was sufficient to achieve feature-learnability performance equivalent to the 87% feature-learnability benchmark. Furthermore, this 2-feature input configuration resulted in no confusion errors under WIA, and very few under LOO conditions, between the tactile and proprioception stimulus categories (i.e., no and few confusion errors in the lower left and upper right quadrants of Figures 5D,E respectively). This result can be explained by the significant difference for *HF spike amplitude* and the >2.5-fold difference in *HF spike count* when tactile-, compared to proprioception-dominated stimuli were used.

For a fixed time-window, *HF spike count* provides information about spike frequency and the total number of action potentials generated by peripheral afferents. The large difference in this feature between tactile- and proprioception-dominated stimuli may have resulted from the relatively small vs. larger receptive field sizes respectively that these stimuli engaged. *HF spike amplitude* captures spiking temporal alignment (as discussed above) and spike magnitudes. Spike magnitudes are influenced by neuron and or axon size, as well as the event’s distance from the electrode (Gasser and Grundfest, 1939; Hunt, 1951; Nelson, 1966; Buchwald and Grover, 1970; Grover and Buchwald, 1970). Group I proprioceptive afferents generally have larger diameters than Aβ tactile afferents (Gasser, 1941). In addition to the laterally placed external cuneate nucleus, proprioceptive afferents preferentially terminate deep on ventral DCN neurons (Campbell et al., 1974), which have a high proportion of large somas (Cheema et al., 1983) that are located 500–700 μm below the brainstem surface in rats (Li et al., 2012). Tactile afferents terminate on smaller neurons in a cluster zone that is located at approximately half the depth as the ventral DCN neurons (Li et al., 2012). How these two neuronal populations (larger somas located deeper vs smaller somas located more superficially) affect spike magnitudes measured from the surface is difficult to assess without dedicated experiments, however, these anatomically segregated neuronal populations likely evoke different shaped spike waveforms. The combination of stimulus-evoked spike-timing, the signal location, and neuronal types responsible for generating the signal may, therefore, result in unique amplitude profiles for each of the stimuli, and thereby contribute to improved stimulus classification.

### Low-Frequency Features

LF features are measures of the rectified signal envelope and therefore capture various aspects of spike bursts. For example, *LF sum burst amplitude*, *LF* *max burst amplitude*, and *LF* *burst count* provide information about burst frequency and magnitudes combined, the largest burst, and the frequency of bursts respectively. The LF feature category demonstrated varied feature-learnability, with its best performer, *LF sum burst amplitudes*, ranking in the top four features in the 1,000 ms and 60 ms windows. This feature likely represents very similar activity to *HF integral*, as both are extracted from rectified HF signals, and summing envelope peaks captures related information to the integral of the signal. Congruently, *LF sum burst amplitudes* and *HF integral* showed very similar feature-learnability characteristics (compare Figures 2A, 6E, 7B,C) and confusion errors (Figures 4A,B), and combining these two features did not significantly improve feature-learnability from *HF integral* alone. These features may capture a combination of HF events, slower synaptic activity, or subthreshold events, representing a measure of the signal energy in a neural population.

### Power Spectral Density Features

The HF PSD feature set was similar to that used by Bouton et al. (2016) who extracted features over 100 ms windows for motor signal decoding in a brain-machine interface capable of effecting limb movement through neuromuscular stimulation. This feature set represents frequency power in the multiunit activity range, which is typically considered to be about 300–6,000 Hz (Stark and Abeles, 2007; our range: 200–4,000 Hz). We found these features generally to be good predictors of somatosensory DCN signals. In our 60 ms window data, the frequency bands of 1.5–2.5 kHz were of interest because: (1) the highest decoding for both tactile- and proprioception-dominated stimuli were found for this feature category over this range; and (2) frequency bands above 1.5 kHz showed a peak in the proprioception-dominated phase, while bands below 1.5 kHz, including the LF PSD features, had peaked in the tactile-dominated phase. Each of the eight HF PSD bands appeared to add some unique information not captured by the other bands, as successive additions of these features continued to improve feature-learnability until all the HF PSD features were exhausted (see the yellow line, Figure 6F). However, feature-learnability derived from all eight HF bands was not significantly greater than combining *HF integral* and *HF spike amplitude*, indicating that these features could offer superior neural decoding compared to all HF PSD features.

### Feature-Learnability Temporal Profiles

Figure 7 provides insight into how neural activity captured by individual features is altered within 60 ms time windows across the dynamic stimuli. The best performing features over a specific period of the stimuli indicates that there are statistically different neural spiking behaviors (Loutit et al., 2019) across the different stimuli for the 60 ms window over which the feature was quantified. Tactile-dominated stimuli evoked small bursts of activity at stimulus onset, while proprioception-dominated stimuli evoked little activity at stimulus onset, but larger amounts during the middle of the stimulus and return periods on anatomically relevant electrodes (Figure 1B). These phases of activity were captured by the peaks of the feature-learnability temporal profile (gold curve, Figure 7A). The 1st peak of this temporal profile reflects the period where considerable tactile information is presented to the ANN because this peak is abolished when tactile data were omitted from the learning algorithm (blue curve, Figure 7A), but remains when tactile data is present and proprioceptive data were omitted (red curve, Figure 7A). The same logic dictates that the 2nd peak is attributed to the duration when maximum proprioceptive information is present. Interestingly, the tactile-dominated feature-learnability temporal profile coincides with the stereotyped characteristics of fast and slowly adapting afferent activity, whereas the proprioception feature-learnability temporal profile is consistent with the dynamic movement of the proprioceptive stimuli, with its peak coinciding when the limbs were maximally flexed or extended (i.e., at the midpoint of the *Move* phase, Figure 7).

The feature-learnability temporal profiles of individual features (Figures 7B–E) provide insight into how significantly different a feature is for the different stimuli (and locations) throughout the progression of the mechanical stimuli (Loutit et al., 2019). HF features capture information about single or multiunit spiking activity, for example, *HF integral*, *HF spike count* and *HF spike amplitude* provide information about signal energy, spiking frequency and amplitude respectively; the latter may relate to the anatomical location relative to the recording site. As LF features capture measures of spike bursts, greater feature-learnability of LF features over the time course of the stimuli indicates the phases of the stimulus where the bursting activity feature is significantly different across the stimuli. For example, LF PSD signal features below 200 Hz do not differ greatly across the proprioceptive-evoked regions of the stimuli, whereas their differences are greater and therefore contribute more toward decoding tactile events. The data suggests that most of the features were able to extract some level of information from single-/multi-unit spikes as well as burst behavior of neural activity during the initial contact of the tactile stimuli, which might reflect the afferent volley of sensory information arriving at the DCN.

### Rest Period Activity

Feature-learnability remained above chance levels 1 s before stimuli were presented (Figure 7) for the *HF spike amplitude* and all HF PSD features. This indicates that during the rest periods, some information specific to the limb and/or the stimulus being presented was encoded in these DCN signal features. That the more successful predictions were not consistent across all animals (Figure 8), suggests that the prediction is not based on some residual activity from particular stimuli, but rather, multiple features in the same animal contributed information that was unique to that animal for a combination of stimulus and location. Furthermore, it was unlikely that above-chance level classification arose from some learned effect during repetitive stimulus presentations because feature-learnability was not different when comparing the first and last third of trials, although, it should be noted that training/testing data sets were also reduced to one third, making accurate classification more challenging.

What then could account for this predictive capacity before the stimuli? One possibility is that the limb undergoing repetitive stimulation experienced a different afferent activation status during the stimulus-off period compared to the remaining limbs. This could have arisen from continued reactivation of slowly adapting afferent activity during the off-stimulus period because of the continued interruption of the repetitive stimulation cycles, whereas the slowly adapting activity from the non-stimulated limbs would have greater opportunity to cease firing. An alternative explanation could be from activity resulting from long-lasting membrane potential depolarizations seen in DCN cells in response to sensory stimulation (Canedo et al., 1998), or rhythmic activity that outlasts stimulation periods (Nuñez and Buño, 1999). We have not found evidence from other studies finding similarly high decoding accuracy in stimulus off periods. Nevertheless, this phenomenon demonstrates remarkable sensitivity of the HF PSD and *HF spike amplitude* features for capturing status differences in afferent populations.

## Conclusion

Feature-learnability enables us to assess the information contained in DCN surface potentials for decoding natural tactile- and proprioceptive-dominated somatosensory events. We identified individual, and combinations of signal features with superior decoding capacity, some of which, to our knowledge, are not currently routinely used for neural decoding. Generally, HF time-domain features are most informative for decoding somatosensory-evoked neural signals compared to frequency-domain features. These features may likely translate to the decoding applications for other neural signals (e.g., motor). For sufficiently large time windows, only two HF time-domain features are adequate to achieve benchmark decoding accuracy, but for shorter time windows that are more practical for real-time applications, increasing the number and diversity of features improves the decoding robustness and accuracy. We showed that a feature’s decoding capacity is altered throughout a dynamic event. Knowledge of how the feature-learnability of different features varies throughout stimulus durations may inform future biomimetic stimulation patterns for a neural prosthesis capable of activating DCN neural populations to restore somatosensory feedback.

## Data Availability Statement

The raw data and extracted features are available here: https://doi.org/10.12751/g-node.jjaaz4.

## Ethics Statement

The animal study was reviewed and approved by Australian National University Animal Experimentation Ethics Committee (A2014/52).

## Author Contributions

JP: conception and design of the study. AL: performed the experiments, data collection and analysis and wrote the first draft of the manuscript. JP and AL: conception of analytical approach and contributed to manuscript revision, read and approved the submitted version.

## Conflict of Interest

The authors declare that the research was conducted in the absence of any commercial or financial relationships that could be construed as a potential conflict of interest.
